# Towards 24/7 MRI: the effect of routine weekend inpatient MRI scanning on patient waiting times

**DOI:** 10.1007/s11845-024-03647-z

**Published:** 2024-03-09

**Authors:** Darragh Garrahy, Simon Doran, Hazel O’Neill, Suzanne Dennan, Peter Beddy

**Affiliations:** Department of Radiology, St James’s Hospital and Trinity College Dublin, James’s St, Dublin 8, Ireland

**Keywords:** Inpatient, MRI, Waiting times, Weekend

## Abstract

**Background:**

Demand for inpatient MRI outstrips capacity which results in long waiting lists. The hospital commenced a routine weekend MRI service in January 2023.

**Aim:**

The aim of this study was to investigate the effect of a limited routine weekend MRI service on MRI turnaround times.

**Methods:**

Waiting times for inpatient MRI scans performed before and after the introduction of weekend MRI from January 1 to August 31, 2022, and January 1 to August 31, 2023, were obtained. The turnaround time (TAT) and request category for each study were calculated. Category 1 requests were required immediately, category 2 requests were urgent and category 3 requests were routine.

**Results:**

There was a 6% (*n* = 128) increase in MRI inpatient scanning activity in 2023 (*n* = 2449) compared to 2022 (*n* = 2322). There was a significant improvement in overall mean TAT for inpatient MRIs (*p* < .001) in 2023 (mean 65.2 h, range 0–555 h) compared to 2022 (mean 98.3 h, range 0–816 h). There was no significant difference in the mean waiting time for category 1 MRIs between 2022 and 2023. There was a significant improvement (*p* < .001) in mean waiting time in 2023 (mean 37.2 h, range 0–555) compared to 2022 (mean 55.4 h, range 0–816) for category 2 MRI. The mean waiting time for category 3 studies also significantly improved (*p* < .001) in 2023 (mean 93.4 h, range 1–2663) when compared to 2022 (mean 154.8, range 1–1706).

**Conclusion:**

Routine weekend inpatient MRI significantly shortens inpatient waiting times.

## Introduction

The demand for medical imaging is unquenchable [1–3]. In Irish publicly funded hospitals, demand for medical imaging outstrips capacity resulting in long waiting lists, this is most pronounced for MRI where the number of scanners in the public system is limited [4]. Delays in performing cross sectional imaging on inpatients result in longer inpatient stays and increase overall cost [5, 6]. A positive correlation has been demonstrated between advanced radiologic imaging, reduced patient length of stay and improved outcome [7]. Increased utilisation of MRI in patients presenting with acute stroke symptoms has been shown to reduce mortality and result in shorter lengths of stay [8].

In the Irish public health system, most hospitals have no emergency MRI service outside the normal working week. Some specialist centres for spinal surgery and neurosurgery have limited emergency out-of-hours MRI access. St James's Hospital is a large acute academic hospital in Dublin city performing 220,000 radiological imaging procedures in 2022. Demand and throughput for advanced medical imaging have risen sharply over the last decade: in 2008, 6850 MRIs were requested and 5000 were completed in St James's Hospital; in 2022, 17,500 were requested and 11,932 completed. The radiology department commenced a weekend MRI service on Sundays in January 2023 to provide improved imaging capacity for both acute emergency requests and routine inpatient imaging.

The aim of this study was to assess the impact of a routine weekend MRI service on patient flow through the radiology department.

## Materials and methods

Ethics approval was granted for this study from the hospital ethics committee. Patient consent was not required.

### Background

The hospital is based in a large urban centre in Ireland and has a local catchment area of 300,000 adults. St James's is also a tertiary referral centre for complex oncology and a major cardiothoracic centre. The radiology department has four MRI scanners, three in the main hospital building (two 1.5 T scanners and one 3 T scanner) and one 1.5 T scanner in the Radiation Oncology centre. The 1.5 T MRI scanners in the main hospital building scan a combination of inpatient and outpatients while the 3 T scans a combination of research study patients and a limited number of outpatients. The scanner in the Radiation Oncology centre scans only outpatients. In 2022, the radiology department performed 8514 MRI scans on outpatients and 3418 scans on inpatients.

### Study design

The study was a retrospective review of inpatient MRI waiting times carried out pre and post the introduction of routine weekend MRI.

### MRI workflow

All inpatient MRI scan requests are placed on the hospital’s electronic patient record (EPR). Each request is reviewed by a radiology specialist registrar or consultant who assigns the request into one of three categories based on the clinical information provided. Category 1 requests are required immediately (e.g. MRI lumber spine for cauda equina), category 2 are urgent (e.g. MRCP to assess for choledocholithiasis) and category 3 are routine (e.g. MRI foot to assess for osteomyelitis). The MRI radiographers review the vetted requests and schedule the scans based on the category associated with the request. During 2022, the MRI scanners operated from Monday to Friday commencing at 0800 and scanning until 1700. From January 1, 2023, the MRI scanners commenced scanning a routine weekend MRI service on a Sunday in addition to the weekday MRI schedule. The weekend service operated from 0800 to 1600 on Sundays. The weekend service scanned all three categories of MRI requests during the operational hours and some urgent outpatient MRI requests.

### Data collection

Waiting times for inpatient MRI scans performed before the introduction of weekend from January 1 to August 31, 2022, and after the introduction of weekend MRI from January 1 to August 31, 2023, were obtained. Each inpatient MRI scan was identified from the hospital business information (BI) system, Microsoft BI. The turnaround time (TAT) for each study was calculated from the time the MRI order was placed on the hospital EPR system to the time the scan was completed on the hospital radiology information system (RIS). The category of each request was also recorded.

### Data analysis

All analyses were carried out in R (version 4.0.1; R Foundation for Statistical Computing, Vienna, Austria). A *p*-value of 0.05 or less was considered statistically significant.

## Results

### Patient demographics

A total of 8646 MRIs inpatient and outpatient studies were performed in the first 8 months of 2022 and 9580 during the first 8 months of 2023 in XYZ hospital. All routine MRI scans performed from January 1 to August 31, 2022, and from January 1 to August 31, 2023, were analysed. There was a 6% (*n* = 128) increase in MRI inpatient scanning activity in 2023 (*n* = 2449) compared to 2022 (*n* = 2322). A total of 721 scans were performed during the weekend imaging sessions in 2023.

### Turnaround times

**Fig. 1:**
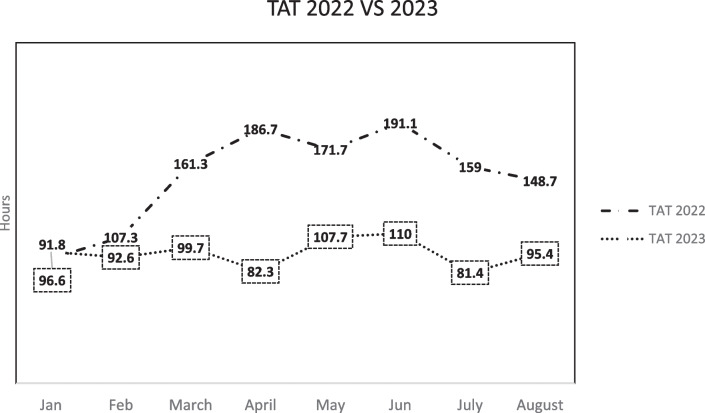
Comparison of turnaround times (TAT) by month for 2022 and 2023

**Fig. 2:**
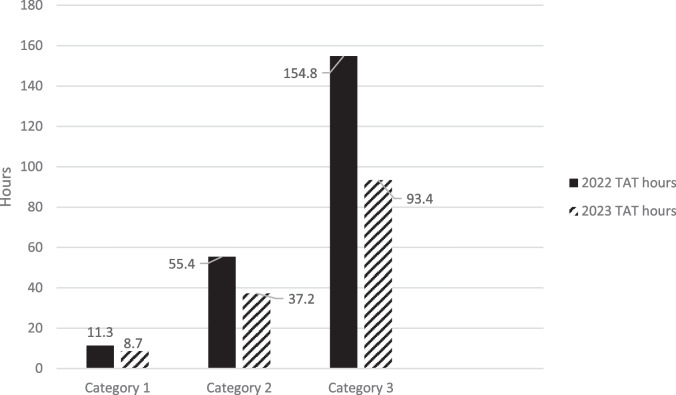
Turnaround times for individual categories of MRI in 2022 and 2023

There was a significant improvement in overall mean TAT for inpatient MRIs (*p* < 0.001) in 2023 (mean 65.2 h, range 0–555 h) compared to 2022 (mean 98.3 h, range 0–816 h) (Fig. 1). In 2022, *n* = 114 scans were vetted as category 1, *n* = 1113 as category 2 and *n* = 1095 as category 3. In 2023, *n* = 116 scans were vetted as category 1, *n* = 980 as category 2 and *n* = 1353 as category 3. There was no significant difference in the number of patients vetted in each category between the two time periods (category 1 *p* = 0.71, category 2 *p* = 0.50, category 3 *p* = 0.43). There was no significant difference (*p* = 0.45) in the mean waiting time for category 1 MRIs in 2023 (mean 8.7 h, range 0–66) compared to 2022 (mean 11.3 h, range 0–263). There was a significant improvement (*p* < 0.001) in mean waiting time in 2023 (mean 37.2 h, range 0–555) compared to 2022 (mean 55.4 h, range 0–816) for category 2 MRI. The mean waiting time for category 3 studies also significantly improved (*p* < 0.001) in 2023 (mean 93.4 h, range 1–2663) when compared to 2022 (mean 154.8, range 1–1706) (Fig. 2).

Ninety-five percent of category 1 scans were performed within 48 h in 2022, and 98% of category 1 scans were performed within 48 h in 2023. Sixty-three percent of category 2 scans were performed within 48 h in 2022 and 77% of category 2 scans were performed within 48 h in 2023. Twenty-four percent of category 3 scans were performed within 48 h in 2022 and 41% of category 3 scans were performed within 48 h in 2023. Forty-two percent of category 3 scans were performed within 96 h in 2022 and 71% of category 3 scans were performed within 96 h in 2023.

## Discussion

Adequate imaging capacity is required to enable clinicians make timely patient management decisions and ultimately to reduce inpatient length of stay [9]. MRI waiting lists are typically the longest of any inpatient imaging modality as scan times are relatively long compared to other imaging modalities such as CT and the number of MRI scanners are limited [4]. In Ireland, most public hospitals operate a weekday-only MRI service with minimal or no out-of-hours capacity. Our study demonstrates that a limited routine weekend inpatient MRI service significantly reduces the waiting times for inpatient scans which is most pronounced for less urgent scans.

The commencement of a weekend MRI service resulted in a relatively modest increase in the overall inpatient MRI activity during the study period (6%). Prior to the weekend MRI lists, the scanners operated for 45 h per week, the weekend lists resulted in an extra 9 h per week of scanning time (20% increase). We did not convert all the potential extra time in to inpatient scanning capacity due to service pressures elsewhere. The ability to scan at weekends allowed the department to slightly reduce the number of inpatient scans performed during weekdays enabling us to scan complex cardiac MRIs and MRIs under general anaesthesia during normal weekday working hours. Despite the relatively modest increase in inpatient scanning numbers from 2022 to 2023 (6%), a 33% reduction in the waiting time for urgent scans was seen and a 40% reduction in the waiting time for routine MRI scans underlining the vast improvement in patient flow through the department. As expected, there was no significant change in the waiting time for scans categorised as immediate. We did not assess the quality of weekend MRI reporting compared to weekday; however, it has previously been shown to be safe [10].

Increased use of MRI for inpatients has been shown to reduce length of stay and lower mortality [8, 9] while early access to MRI is important as it significantly reduces a patient’s overall length of stay [7]. Patients admitted over weekends have been shown to wait significantly longer for MRI when compared to those admitted during the week which likely relates to limited access to routine MRI [11]. MRI scanners that operate on a weekday-only service are underutilised; a scanner operating from 0800 to 1700 each weekday will result in 2250 h of scanning per year (based on 250 working days per annum). If a scanner operated on a 24-h basis for 365 days per annum (24/7 service), this would give 8760 scanner hours. Most MRI scanners in the public health system in Ireland run for approximately 26% of their potential operational hours. Our study demonstrated that a modest increase in inpatient scanning capacity can result in a very significant reduction in inpatient waiting times. If scanners operated at full capacity, inpatient waiting lists could potentially be eliminated.

There are many barriers to establishing a 24/7 MRI service in a publicly funded health system, primarily the lack of MRI trained staff, limited reporting capacity and strict labour laws [12]. Training an MRI radiographer to work independently can take up to 1 year after commencement of MRI placement. Once trained, retention of MRI radiographers is problematic. The private sector can attract staff with preferential on-call rates including fee-per-item scanning payments. Many younger staff also seek opportunities to work abroad where MRI-trained radiographers are in high demand. Our hospital is also based in an urban centre where rents and house prices are very challenging for staff. There is also a national shortage of radiologists in Ireland with consultant numbers of 5.8 per 100,000 population, significantly below the OECD average of 8 radiologists per 100,000 [13–15]. Low consultant numbers limit the reporting capacity to take on initiatives such as weekend MRI. Despite these challenges, most large academic hospitals have established CT on call rosters which enable 24/7 scanning, and we should aim to expand this to MRI.

The study has several limitations. It was a single-centre study and the findings may not be applicable to other sites where the case mix and subspecialty practice is different. We made no attempt to assess the direct impact of weekend MRI on length of stay as this is a complex and multifactorial issue not entirely dependent on radiology imaging turnaround; we plan to study this further during the continued roll-out of the service. The additional imaging capacity was not solely used to improve patient flow, and routine inpatient waiting time improvements may have been underestimated as we used some of the additional capacity to increase our capacity for complex outpatient imaging.

In conclusion, routine weekend inpatient MRI significantly shortens waiting times, improves patient flow and may shorten length of stay despite only modestly increasing the overall number of MRIs performed.
